# Deposition and Properties of Amorphous TiBN/AlCrYN Multilayer Coatings with Various Modulation Periods

**DOI:** 10.1155/2020/3758209

**Published:** 2020-07-17

**Authors:** Wei Dai, Fan Liu, Qimin Wang

**Affiliations:** School of Electromechanical Engineering, Guangdong University of Technology, Guangzhou 510006, China

## Abstract

In this paper, multilayer coatings consisted of amorphous AlCrYN layers and TiBN layers were deposited by the cosputtering technique. The influence of the modulation period of the multilayer coatings on the structure, mechanical properties, and oxidation behavior of the coatings was studied carefully by using scanning electron microscope, X-ray diffraction, nanoindentation, scratch tester, and thermogravimetric analyzer. The results show that the TiBN/AlCrYN multilayer coatings exhibit an amorphous structure without any feature. Decreasing the modulation period could significantly improve the coating hardness and elastic modulus. In addition, the adhesion of the multilayer coatings could be enhanced as the modulation period decreases. At relative low oxidation temperature (≤900°C), a dense aluminum oxide layer formed on the coating surface can effectively hinder the inward diffusion of O and the outward diffusion of metal elements. The oxidation behaviors of the TiBN/AlCrYN multilayer coatings obeyed the diffusion control law. The oxidation resistance of the coatings was increasing with decreasing the modulation period since the interfaces of multilayer structures would block the diffusion of elements. At relative high oxidation temperature (1000°C), however, the coating surface was rapidly oxidized into the porous TiO_2_ whiskers rather than the dense Al_2_O_3_ layer, resulting in the inward diffusion of O and thus causing the serious oxidation of the coatings.

## 1. Introduction

Hard nitride coatings deposited by physical vapor deposition (PVD) are widely used as protective coatings in many applications, such as the protection of cutting tools and molds due to their high hardness and wear resistance [1–3]. However, the thermal stability, toughness, and adhesion of the hard nitride coatings are still strong challenges for the increasing industrial applications especially under extreme conditions, e.g., high temperature [4]. Unfortunately, the PVD hard nitride coatings usually present columnar growth structures where oxygen is able to diffuse into the coatings while metal elements diffuse towards the coating surface through the columnar grain boundary, resulting in serious oxidation of the PVD coatings [5]. In addition, some unavoidable “through-thickness defects” like micropits and micropores in the PVD coatings can also provide channel for the diffusion of oxygen and thus enhance the oxidation of the coatings [5]. On the other hand, the hard coatings prepared by PVD process usually have high residual stress, causing the poor adhesion to the substrate. Now, therefore, it is highly desirable to improve the oxidation resistance and adhesion of the PVD coatings.

Amorphous phase usually possesses the smooth dense structure without grain boundaries, which would effectively avoid boundary diffusion. On the other hand, multilayered architectural structures have been demonstrated to significantly enhance the mechanical properties and oxidation resistance of the PVD coatings [6, 7]. The interfaces among heterolayers can effectively restrain the crack growth and block the diffusion of oxygen and other media corrosion across the PVD coatings and thus improve the oxidation resistance, corrosion resistance, and mechanical properties of PVD coatings [7]. Accordingly, it is supposed that combining the multilayered architectural structure with the amorphous phase structure can significantly improve the oxidation resistance of the hard PVD coatings.

Previous literature reports that AlCrYN coatings exhibit high oxidation resistance at high temperature due to the formation of compact oxide layer on the surface [8]. In this paper, multilayer coatings consisted of amorphous AlCrYN layers and TiBN layers were deposited by the cosputtering technique. The influence of the modulation period of the multilayer coatings on the structure, mechanical properties, and oxidation behavior was studied carefully. Furthermore, the relationships between the deposition process, microstructure, and properties of the coatings were discussed carefully.

## 2. Experimental Details

A direct current magnetron cosputtering deposition system composed of two independent magnetron sputtering units was employed to prepare the TiBN/AlCrYN multilayer coatings. Pure TiB_2_ (99.95%, Ti : B = 33 : 67 at.%) and AlCrY (99.97%, Al : Cr : Y = 67 : 30 : 3 at.%) rectangular planar with the length of 50 mm and width of 480 mm were used as the sputtering targets. Polished cemented carbide (WC with 6 wt.% Co, mirror polish) and polycrystalline alumina wafers were used as the substrates for the characterization of mechanical properties and the high temperature oxidation test, respectively. All the substrates were cleaned each sequentially by using ultrasonic baths of acetone and alcohol for 20 minutes. The based pressure in the vacuum chamber was below 5 × 10^−3^ Pa. Ar glow discharge cleaning with -650 V applied on the substrate holder was taken for 3 minutes to remove the impurities and oxide on the substrate surface before deposition. During coating process, a gas mixture of Ar and N_2_ (Ar/N_2_ = 1 : 1) with 100 sccm was input into the chamber. The deposition pressure was kept around 0.6 Pa. The sputtering powers of the TiB_2_ target and AlCrY target were maintained at 2 kW. The bias voltage of -100 V was applied on the substrate. The deposition temperature was about 350°C. The total deposition duration was 4 hours, and the thicknesses of all coatings were kept around 1.2 *μ*m. The distances are approximately 50 mm between the sputtering targets and the substrate holder. The substrate holders were placed on a central rotary table. The modulation period of the multilayered TiBN/AlCrYN coatings was adjusted by varying the rotation speed of the central rotary table from 0.5 rpm to 3 rpm.

The growth morphologies of the coatings were observed by scanning electron microscopy (SEM, FEI, Nano430) with an operating voltage of 10 kV. The phase structure of the as-deposited coatings was characterized by X-ray diffraction (XRD, Bruker D8 Advance diffractometer, CuK*α*). The scanning angle of the 2*θ* ranged from 20° to 80° with a step width 0.02° at a speed of 2°/min. The coating hardness and elastic modulus were measured using a nanoindentation tester (CSM, TTX-NHT) with a Berkovich diamond indenter under the constant load of 5 mN. The indentation depth was controlled to about 10% of the coating thickness to minimize the effect of the substrate. The scratch test was performed by a Nano Scratch Tester (CSM, Revetest scratch tester) using Rockwell C diamond styli with a radius 200 *μ*m to evaluate the adhesion strength of the coatings. The scratch length was 3 mm, along with the normal load increased from 1 N to 100 N with a scratch speed of 6 mm/min. The scratch morphologies of the scratch tracks were performed by an optical microscope. A thermal gravimetric (TG) analyzer (Setsys TMA, France) was used to investigate the oxidation behavior of the as-deposited coatings which were heated up to 800°C, 900°C, and 1000°C in oxygen atmosphere with a heating rate of 10°C/min for two hours. The samples for the TG test were deposited on the polycrystalline alumina substrates. The surface morphology and composition of the coatings after the oxidation test were investigated by SEM and energy-dispersive X-ray detector (EDS, FEI, Nano430) with an operating voltage of 20 kV and emission current of 10 *μ*A.

## 3. Results and Discussion

**Figure 1:**
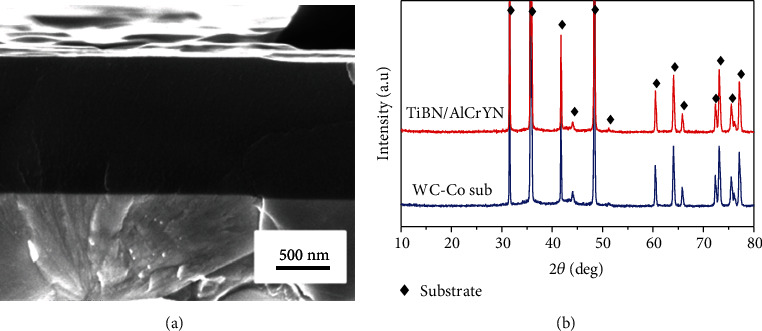
(a) Typical cross-sectional SEM image and (b) XRD pattern of the coating deposited with the rotation speed of 2 rpm.

Figure 1(a) shows the typical cross-sectional morphology of the TiBN/AlCrYN multilayer coating deposited with the rotation speed of 2 rpm. It can be seen that the coating shows dense and smooth glass-like morphology without any feature. The corresponding XRD diffractogram (Figure 1(b)) illustrates that there are no diffraction peaks attributed to the coating, which also confirm the amorphous structure of the coating. The formation of the amorphous structure might be related to the low modulation period of the coatings. The coating modulation period calculated through dividing the total coating thickness by the rotation numbers of the central rotary table decreases from 12 nm to 2 nm as the rotation speed of table increases from 0.5 rpm to 3 rpm. This means that the individual layers (both TiBN layers and AlCrYN layers) are very thin and hard to crystallize since the multilayered structure would inhibit the growth of the crystalline grains.

**Figure 2:**
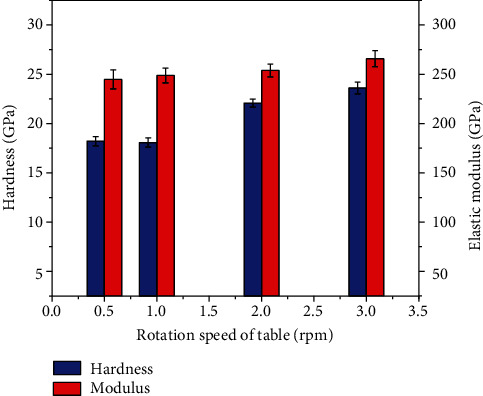
Hardness and elastic modulus of the TiBN/AlCrYN multilayer coatings with various rotation speeds of table.

Figure 2 exhibits the hardness and elastic modulus of the coatings deposited with various the rotation speeds of the substrate holder table. It can be seen that the hardness of the coatings increases from 16 GPa to 22 GPa as the rotation speed of table increases from 0.5 rpm to 3 rpm. It is reported that decreasing the thicknesses of the individual layers in the multilayered structure system would enhance the coating hardness due to the complex effects of interface structures among heterolayers [9]. Additionally, decreasing the modulation period could effectively refine the microstructure of the coating components and thus improve significantly the coating mechanical properties.

**Figure 3:**
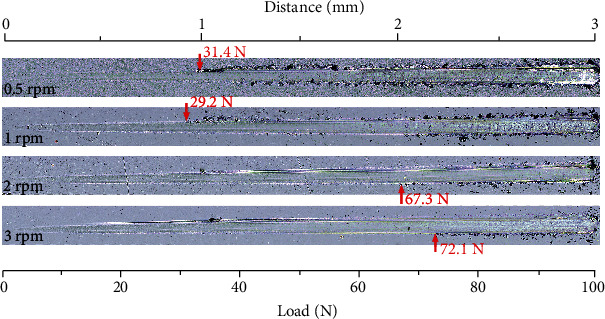
Scratch morphologies of the TiBN/AlCrYN multilayer coatings deposited with different the rotation speeds of table.

Figure 3 exhibits the typical scratch morphologies of the TiBN/AlCrYN multilayer coating deposited with different rotation speeds of table. The coating deposited with 0.5 rpm shows a relatively low critical load of about 31.4 N. It should be noted that cracking and fragmentation occur prior to the onset of substrate exposure. The appearances of cracking and fragmentation might be attributed to the severe plastic deformation of the substrate under high load. The brittle hard coating cannot adapt to the deformation and thus tends to crack when being depressed into the substrate. Meanwhile, the fragmentations would peel off due to low adhesive strength of the coating/substrate. As the modulation period of the coatings decreases (the rotation speed of table increases), however, the critical load increases significantly. The critical load of the TiBN/AlCrYN multilayer coating increases to 72.1 N when the rotation speed of table increases to 3 rpm. Although the cracks also appeared before the onset of substrate exposure, they became very small and no fragmentation was observed to peel from the substrate, implying the good scratch resistance and high adhesion strength of the coatings.

The enhancement of the scratch resistance of the coatings might be ascribed to the multilayered structure where the interfaces between TiBN/AlCrYN layers can effectively prevent the propagation and extension of cracks and thus greatly improve the toughness of the TiBN/AlCrYN multilayer coatings [10]. Additionally, according to the results of nanoindention (Figure 2), the hardness/elastic modulus ratio (*H*/*E*) of the coatings increases from 0.07 to 0.09 as the rotation speed of table increases from 0.5 rpm to 3 rpm. Usually, the *H*/*E* is expected to correlate with the elastic resilience and a high *H*/*E* implies that the coating has a high elastic strain prior to the plastic deformation [11]. It is clear that the high *H*/*E* value is beneficial to the scratch resistance. As a result, the scratch resistance of the TiBN/AlCrYN multilayer coatings increases as the modulation period decreases (the rotation speed of table increases). Furthermore, the multilayered structure is also believed to effectively reduce residual stress and then enhance the coating adhesion.

**Figure 4:**
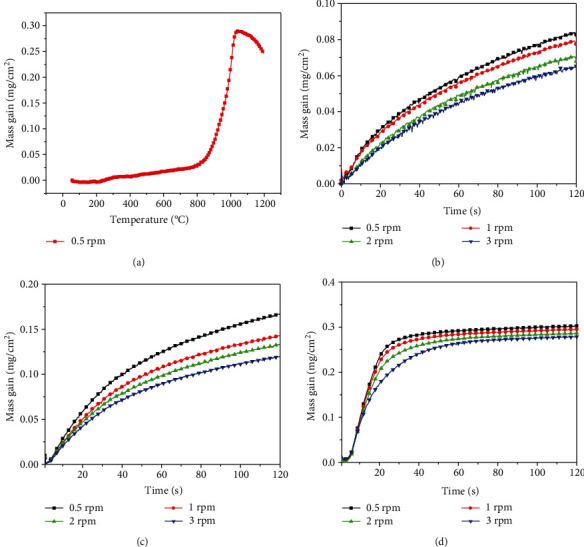
(a) Typical thermogravimetric (TG) curve of the coating deposited with 0.5 rpm and TG curves of the coatings during isothermal oxidation at (b) 800°C, (c) 900°C, and (d) 1000°C.

The oxidation behaviors of the coatings were performed by TG as shown in Figure 4.  Figure 4(a) presents the typical TG curve of the coating deposited with 0.5 rpm. The mass gain of the coating increases slightly with the temperature at initial stage of heating (200°C~800°C), followed by a significant increase of the mass gain at the temperatures of about 800°C. However, the mass gain of the coating shows a significant decrease when the temperature exceeds 1000°C. In order to insight into the oxidation behaviors of the coatings, isothermal TG of the TiBN/AlCrYN multilayer coatings with different modulation periods was taken at temperatures of 800°C, 900°C, and 1000°C. Figures 4(b)–4(d) exhibit the mass grains of the coatings as a function of the oxidation time. It can be seen that the mass grains of all the samples increase with the oxidation time at all temperatures, but the shapes of the curves are different. Under the oxidation temperatures of 800°C (Figure 4(b)) and 900°C (Figure 4(c)), the mass gains of the coatings increase slowly and exhibit parabolic oxidation curves. However, the mass gain curves of the coatings could be divided into two different parts when the oxidation temperature increases to 1000°C (Figure 4(d)). At initial stage (<20 min), the mass gain of the coating increases rapidly and occurs linearly. Subsequently, the mass gains of the coatings exhibit parabolic rate. It should be noted that the mass gains of the coatings decrease as the modulation period of the coatings decreases (the rotation speed of table increases) at certain oxidation time.

**Figure 5:**
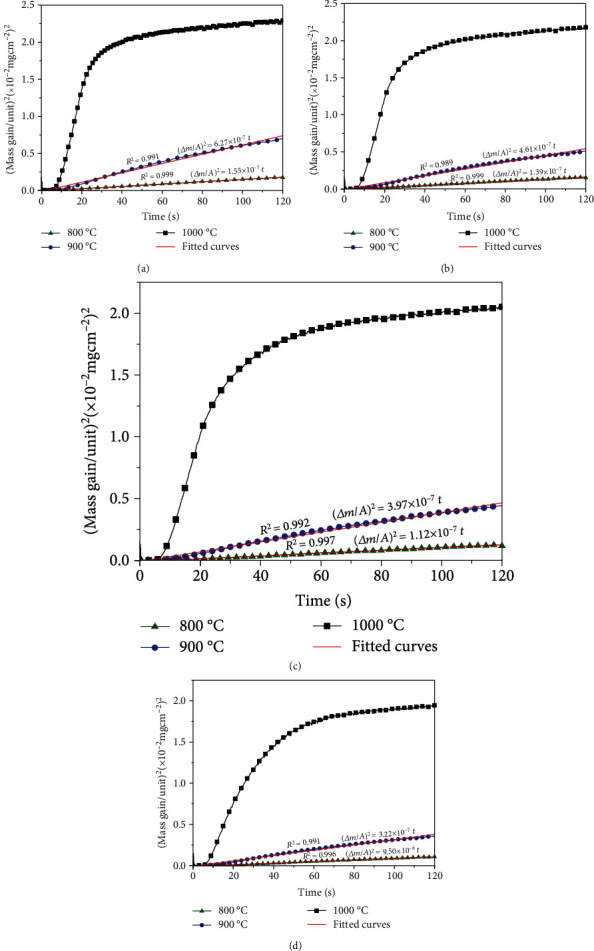
Plots of (Δ*m*/*A*)^2^ versus time for the TiBN/AlCrYN coatings with different rotation speeds of (a) 0.5 rpm, (b) 1 rpm, (c) 2 rpm, and (d) 3 rpm between 800°C and 1000°C. The best-fitting straight lines are given with the coefficient of determination *R*^2^ in the plots.

The parabolic oxidation rate has been expected to follow diffusion control law which can be expressed by the equation of (Δ*m*/*A*)^2^ = *kt* + *c*, where the Δ*m* is the mass change, *t* is time, *A* is the surface area of the sample, and *k* and *c* are the parabolic rate constant and constant, respectively [12].  Figure 5 presents the curves of (Δ*m*/*A*)^2^ versus *t* and the fitted straight lines for the date points. The coefficient values of determination (*R*^2^) which indicate the goodness of fit and *k* values are also included in the figures. It can be seen that the *R*^2^ values of all the samples are almost higher than 0.99 at 800°C and 900°C, indicating that all curves accord with the parabolic law. This illustrates that the oxidation reaction of the TiBN/AlCrYN multilayer coatings was controlled by diffusion process at relatively low temperature (≤900°C). It should be noted that *k* values at 900°C are higher than that at 800°C, since the oxidation rate increases with temperature. Furthermore, the *k* value decreases significantly as the rotation speed of table increases. In addition, the activation energy for oxidation of the coatings calculated according to the Arrhenius equation increases from about 125 kJ mol^−1^ to 185 kJ mol^−1^ [12]. This indicates that decreasing the modulation period of the coatings would enhance the coating oxidation resistance. However, it can be seen that the oxidation behavior does not follow the parabolic law and the coating exhibits the high oxidation rate when the oxidation temperature is 1000°C.

**Figure 6:**
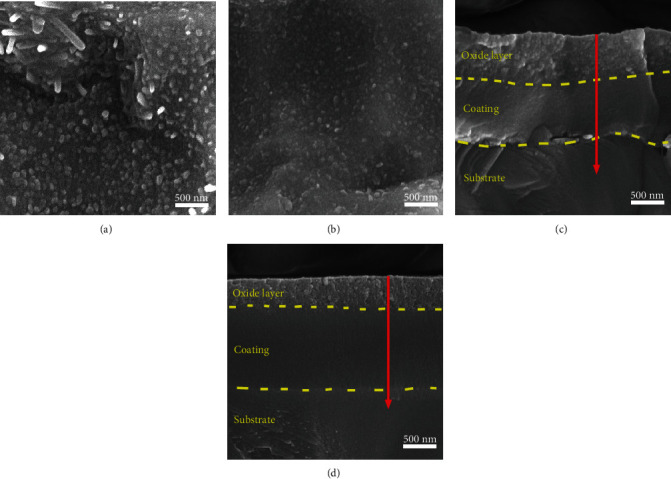
Typical surface and cross-sectional morphologies for the TiBN/AlCrYN multilayer coatings deposited with the rotation speeds of 0.5 rpm (a, c) and 2 rpm (b, d) after oxidation at 900°C.

**Figure 7:**
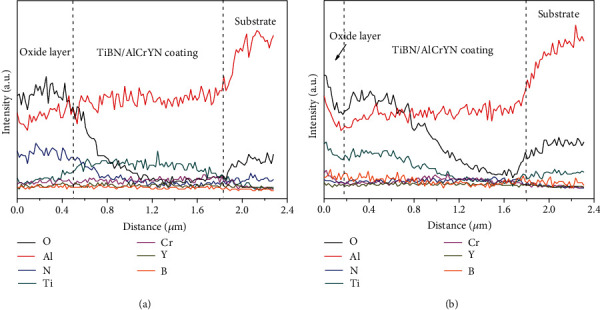
(a, b) The corresponding EDS line-scanning profiles along the red arrows shown in Figures 6(c) and 6(d), respectively.

The typical surface topographies and cross-sectional morphologies of the coatings deposited with 0.5 rpm and 2 rpm after oxidation at 900°C are shown in Figure 6. It can be seen that the coating deposited with 0.5 rpm (Figure 6(a)) shows numerous granular and rod-like features on the surface. The corresponding cross-sectional SEM image reveals that a dense oxidation layer with a thickness of about 500 nm appears on the surface of the coating. As the rotation speed of table increases to 2 rpm, the coating shows a significant decrease on the size and quantity of the granular features on the surface. And the thickness of the oxidation layer decreases to about 200 nm. This also proves that the coating with the relative low modulation period shows a high oxidation resistance. It is supposed that the interfaces of multilayer structure would block the diffusions and thus enhance the oxidation resistance of the coatings. The EDS line-scanning profiles along with the coating thickness (red arrows in Figures 6(c) and 6(d)) are presented in Figure 7. It can be seen that the oxide layers on the coating surfaces mainly contain Al and O, implying that the oxide layer is consisted of aluminum oxides. The dense aluminum oxide layer could effectively hinder the inward diffusion of O and the outward diffusion of metal elements [13]. Accordingly, the oxidation behavior of coatings obeys the diffusion control law at the oxidation temperature of 900°C.

**Figure 8:**
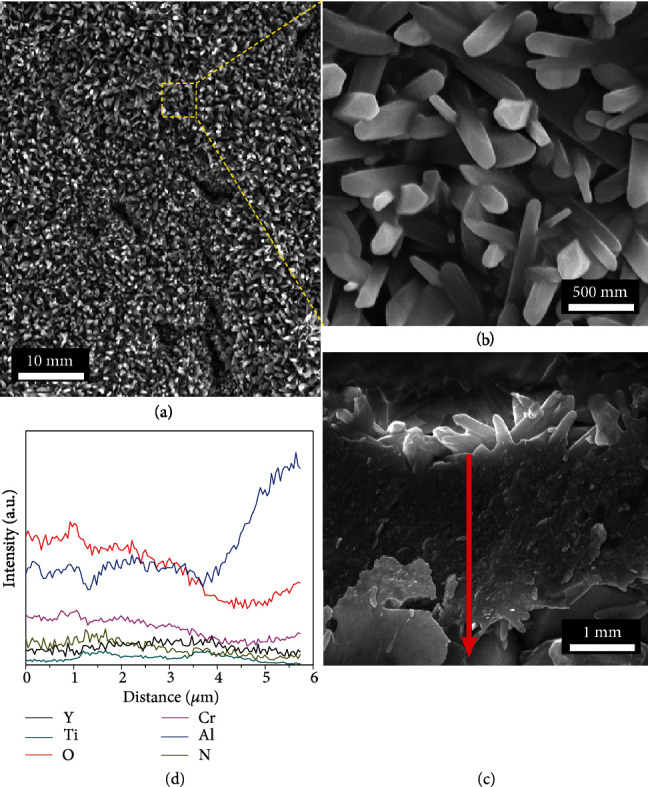
(a) Surface morphology, (b) enlarged view of (a), (c) cross-sectional morphology, and (d) the corresponding EDS line-scanning profiles along the red arrow shown in (c) of the TiBN/AlCrYN multilayer coating deposited with the rotation speeds of 0.5 rpm after oxidation at 1000°C.

Figure 8 gives the oxidation morphologies of the coating deposited with 0.5 rpm at the oxidation temperature of 1000°C. It can be seen that numerous sharp whiskers that can be ascribed to TiO_2_ are observed on the coating surface, implying oxidizing quickly of the coating in the initial stage of oxidation. The EDS line-scanning profiles (Figure 8(d)) along with the coating thickness (red arrow in Figure 8(c)) illustrate that oxygen exists throughout the coating, indicating that the coating was almost completely oxidized. The coating thickness after oxidation also increased greatly due to the volume expansion of oxide. The cross-sectional image of the coating reveals that the oxide layer is consisted of two layers (Figure 8(c)). The layer on the top is the porous whiskers, followed by a dense oxide layer. According to the results above, the mass gain curve of the coating at the oxidation temperature of 1000°C (Figure 4(d)) can be divided into two different parts. In the initial stage of oxidation, the surface layer was oxidized rapidly into the porous TiO_2_ whiskers due to the high oxidation temperature (1000°C). The TiO_2_ porous whiskers hinder the formation of the dense oxide layer and allow the inward diffusion of O. As a result, the coatings showed a high oxidation rate at initial stage (<20 min) of the isothermal oxidation test (as shown in Figure 4(d)). Subsequently, the dense oxide layer was formed in the coatings, which could act as a barrier layer and block the element diffusion. Accordingly, the mass gains of the coatings exhibit parabolic rate after the initial stage.

## 4. Conclusions

Amorphous TiBN/AlCrYN multilayer coatings with different modulation periods were deposited by the cosputtering technique through varying the rotation speed of table. The effect of the modulation period on the structure, mechanical properties, and oxidation behavior of the coatings was studied carefully. It is found that the amorphous TiBN/AlCrYN multilayer coatings exhibit the glass-like morphology without any feature. The hardness and adhesion of the coatings could be greatly improved via decreasing the modulation period. The multilayer coatings show the high oxidation resistances at relative low oxidation temperature (≤900°C). The enhancement of the oxidation resistance could be attributed to the formation of the dense aluminum oxide layer which can effectively hinder the diffusion of elements, causing the oxidation behavior of coatings to follow the diffusion control law. However, as the oxidation temperature increases further (1000°C), the coating surface would be rapidly oxidized into the porous TiO_2_ whiskers, which prevented the formation of the dense oxide layer and allowed the inward diffusion of O, resulting in a serious oxidation of the coatings. Decreasing the modulation period could be conducive to improving the coating oxidation resistance since the interfaces of multilayer structure would block the diffusions.

## Data Availability

The data used to support the findings of this study are available from the corresponding author upon request.

## References

[1] Bobzin K. (2017). High-performance coatings for cutting tools. *CIRP Journal of Manufacturing Science and Technology*.

[2] Inspektor A., Salvador P. A. (2014). Architecture of PVD coatings for metalcutting applications: a review. *Surface and Coating Technology*.

[3] Tkadletz M., Schalk N., Daniel R., Keckes J., Czettl C., Mitterer C. (2016). Advanced characterization methods for wear resistant hard coatings: a review on recent progress. *Surface and Coating Technology*.

[4] Musil J. (2012). Hard nanocomposite coatings: Thermal stability, oxidation resistance and toughness. *Surface and Coatings Technology*.

[5] Panjan P., Cekada M., Panjan M. (2012). Surface density of growth defects in different PVD hard coatings prepared by sputtering. *Vacuum*.

[6] Xiao B., Li H., Mei H. (2018). A study of oxidation behavior of AlTiN- and AlCrN-based multilayer coatings. *Surface and Coating Technology*.

[7] Dai W., Wang Q., Kim K. H., Kwon S. H. (2019). Al_2_O_3_/CrAlSiN multilayer coating deposited using hybrid magnetron sputtering and atomic layer deposition. *Ceramics International*.

[8] Rojas T. C., Dominguez-Meister S., Brizuela M., Sanchez-Lopez J. C. (2019). Influence of Al and Y content on the oxidation resistance of CrAlYN protective coatings for high temperature applications: new insights about the Y role. *Journal of Alloys and Compounds*.

[9] Wang Y. X., Zhang S. (2014). Toward hard yet tough ceramic coatings. *Surface and Coating Technology*.

[10] Carvalho N. J. M., De Hosson J. T. M. (2006). Deformation mechanisms in TiN/(Ti,Al)N multilayers under depth-sensing indentation. *Acta Materialia*.

[11] Charitidis C. A. (2010). Nanomechanical and nanotribological properties of carbon-based thin films: a review. *International Journal of Refractory Metals and Hard Materials*.

[12] Ding Y., Hussain T., McCartney D. G. (2015). High-temperature oxidation of HVOF thermally sprayed NiCr-Cr_3_C_2_ coatings: microstructure and kinetics. *Journal of Materials Science*.

[13] Xu X. Y., Riedl H., Holec D., Chen L., Du Y., Mayrhofer P. H. (2017). Thermal stability and oxidation resistance of sputtered Ti Al Cr N hard coatings. *Surface and Coatings Technology*.

